# Association of growth and nutritional parameters with pulmonary function in cystic fibrosis: a literature review

**DOI:** 10.1016/j.rppede.2016.02.001

**Published:** 2016

**Authors:** Renan Marrichi Mauch, Arthur Henrique Pezzo Kmit, Fernando Augusto de Lima Marson, Carlos Emilio Levy, Antonio de Azevedo Barros-Filho, José Dirceu Ribeiro

**Affiliations:** Faculdade de Ciências Médicas, Universidade Estadual de Campinas (Unicamp), Campinas, São Paulo, Brazil

**Keywords:** Cystic fibrosis, Lung/pathophysiology, Growth, Nutrition

## Abstract

**Objective:**

To review the literature addressing the relationship of growth and nutritional parameters with pulmonary function in pediatric patients with cystic fibrosis.

**Data source:**

A collection of articles published in the last 15 years in English, Portuguese and Spanish was made by research in electronic databases - PubMed, Cochrane, Medline, Lilacs and Scielo - using the keywords cystic fibrosis, growth, nutrition, pulmonary function in varied combinations. Articles that addressed the long term association of growth and nutritional parameters, with an emphasis on growth, with pulmonary disease in cystic fibrosis, were included, and we excluded those that addressing only the relationship between nutritional parameters and cystic fibrosis and those in which the aim was to describe the disease.

**Data synthesis:**

Seven studies were included, with a total of 12,455 patients. Six studies reported relationship between growth parameters and lung function, including one study addressing the association of growth parameters, solely, with lung function, and all the seven studies reported relationship between nutritional parameters and lung function.

**Conclusions:**

The review suggests that the severity of the lung disease, determined by spirometry, is associated with body growth and nutritional status in cystic fibrosis. Thus, the intervention in these parameters can lead to the better prognosis and life expectancy for cystic fibrosis patients.

## Resumo

**Objetivo:**

Revisar a literatura que aborda a relação entre os parâmetros de crescimento e nutricionais com a função pulmonar em pacientes pediátricos com fibrose cística.

**Fontes de dados:**

Dados foram coletados de artigos publicados nos últimos 15 anos em Inglês,Português e Espanhol através de pesquisa nas bases de dados eletrônicas - PubMed, Cochrane, Medline, Lilacs e Scielo - usando as palavras-chave: fibrose cística, crescimento, nutrição, função pulmonar utilizando combinações variadas. Os artigos que analisaram a associação de longo prazo entre parâmetros de crescimento e nutricionais, com ênfase em crescimento, com doença pulmonar em fibrose cística, foram incluídos, sendo excluídos aqueles que analisaram apenas a relação entre os parâmetros nutricionais e fibrose cística e aqueles em que o objetivo era descrever a doença.

**Síntese dos dados:**

Sete estudos foram incluídos, com um total de 12.455 pacientes. Seis relataram relação entre parâmetros de crescimento e função pulmonar, incluindo um estudo que analisou apenas a associação de parâmetros de crescimento com a função pulmonar, e todos os sete relataram associação entre parâmetros nutricionais e função pulmonar.

**Conclusões:**

A revisão sugere que a gravidade da doença pulmonar, determinada por espirometria, está associada com crescimento corporal e o estado nutricional em fibrose cística. Assim, a intervenção nesses parâmetros pode contribuir para um melhor prognóstico e expectativa de vida em pacientes com fibrose cística.

## Introduction

Cystic Fibrosis (CF) is the most common lethal genetic disease in Caucasian populations. It is caused by a mutation in a gene that encodes the Cystic Fibrosis Transmembrane conductance Regulator (CFTR) protein, which is expressed in many epithelial and blood cells, functioning mainly as a chloride channel.^1^ Pulmonary disease is the most important manifestation in CF and the main factor acting in morbidity and mortality of the disease. The response in pulmonary disease is mediated by abnormal CFTR,^2^ modifier genes,^3^^-^ 8 airway infections and inflammation,^9^ probably affecting weight and height due to appetite suppression and enhanced energy expenditure.

Malnutrition and growth restriction are also frequent and are related to the impairment of the pulmonary function in a vicious circle: malnourished patients tend to present worst pulmonary function and patients with severe pulmonary disease tend to grow up less. Although these relationships have been already reported,^10^^,^ 11 there are a few long term analyses in regard to the achievement of growth and nutrition goals for the course of pulmonary function from infancy to adulthood.

In this context, the aim of this study was to analyze long term studies comparing growth and nutrition parameters (with emphasis in growth) with pulmonary function in CF patients, evaluating the relationship among these factors.

## Method

A literature review of the last 15 years (2000-2015) about the relationship between growth and nutritional parameters and lung function was made. The search for references in English, Spanish and Portuguese was performed through electronic databases - PubMed, Medline, Cochrane, Lilacs and Scielo - using the descriptors: CF, growth, body growth, pulmonary function and lung function in varied combinations and in their correspondent translations to Portuguese and Spanish. Reviews addressing the theme were also consulted, as well reference lists of all articles, to search for new studies.

After this stage, we started the screening of papers, by analyzing titles and abstracts. The first inclusion criterion was the identification of potentially relevant studies, considering those in which the report compared growth parameters with lung function. In this case, we excluded studies in which the aims were to compare weight and/or height gain, without relationship with pulmonary function, and those in which the aims were to describe CF only.

**Figure 1:**
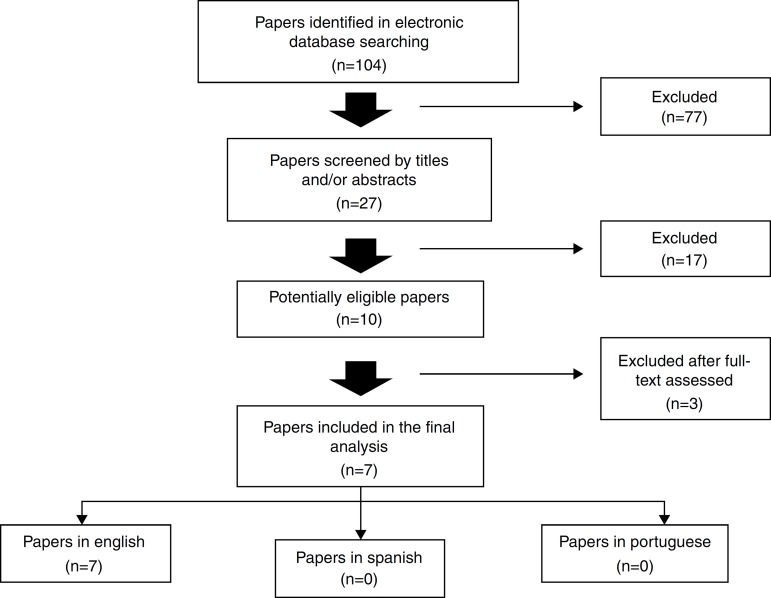
Method of research, screening, exclusion and inclusion of papers in the final analysis. No papers in Spanish and Portuguese were found.

In the first search, a total of 104 articles were found. By evaluating titles and abstracts, the following recuperation criteria for complete articles were: studies of cohort, longitudinal, cross-sectional, descriptive and prospective, which results evaluated the relationship among pulmonary function and growth parameters in CF patients, excluding those which, despite of appearing in the search results, did not address the subject under this point of view. In this stage, 27 papers were screened. The review was concluded with the reading of the complete articles, and, in the final manuscript, seven articles were included,^12^^-^ 18 all of them in English (Fig. 1; Tables 1 and 2).

**Table 1:** Description of the studies included in the systematic review published before 2010, distributed by authorship, design, location, objective or hypothesis, main variables studied and main findings.

Study	Design	Location	Objective	Variables analyzed	Main findings
Zemel et al. (2000)^12^	Cohort	Philadelphia, United States	To determine the relationship among growth, nutritional status and pulmonary function in cystic fibrosis patients over a 4 year period	Forced Expiratory Volume in 1 second (Percentage of predicted), Height for Age Z score, Weight for Age Z score, Body Mass Index, percentage of height-appropriate Body Weight	The *Z*-scores for weight and percentage of height-appropriate Body Weight were associated with longitudinal changes in FEV1% after adjustments was done for hospitalizations
Konstan et al. (2003)^13^	Longitudinal	Multicentric (United States and Canada)	To examine the relative roles of growth and nutritional status and clinical evidence of lung disease from age 3 to 6 years in determining pulmonary function at age 6 years	Weight For Age, Height for Age, Percentage of Ideal Body Weight, Body Mass Index, Forced Expiratory Volume in 1 second, Forced Vital Capacity	Weight For Age, Height For Age and Percentage of Ideal Body Weight were poorly associated with lung disease at age 3, but all were strongly associated with pulmonary function at age six
Peterson et al. (2003)^14^	Longitudinal	Minneapolis, United States	To evaluate how the weight gain pattern of children with cystic fibrosis affects their pulmonary function development	Baseline height; height gain; weight gain	Children who had steady weight gain tended to experience greater increases in Forced Expiratory Volume in 1 second than children who experienced periodic losses in weight
Assael et al. (2009)^15^	Longitudinal	Veneto Region, Italy	To find a correlation between growth and lung disease severity through childhood	Forced Expiratory Volume in 1 second, height gain	Lung disease severity correlated with delayed prepubertal and pubertal growth milestones. Peak height velocities were reduced in relation to the severity of the disease

**Table 2:** Description of the studies included in the systematic review published after 2010, distributed by authorship, design, location, objective or hypothesis, main variables studied and main findings.

Study	Design	Location	Objective	Variables analyzed	Main findings
Yen et al. (2013)^16^	Prospective	Multicentric (United States)	To evaluate the relationship between nutritional status early in life and the timing and velocity of height growth, lung function, complications of cystic fibrosis, and survival	Body Mass Index, Forced Expiratory Volume in 1 second, Height for Age Percentile, Weight for Age Percentile	Weight for Age Percentile>10% at age 4 was associated with better lung function from ages 6-18. By age 18, patients with an age 4 WAP>50% suffered fewer acute pulmonary exacerbations
Woestenenk et al. (2014)^17^	Longitudinal	Utrecht, The Netherlands	To measure the weight and height of children with cystic fibrosis from 2 to 10 years old and to investigate the relationship between these parameters and Forced Expiratory Volume in 1 second beginning at 6 years old	Forced Expiratory Volume in 1 second, Weight For Age, Height For Age, Height For Age (adjusted for Target Height), Weight For Height	The yearly decline in Forced Expiratory Volume in 1 second was significantly slowed in children who gained weight
Sanders et al. (2015)^18^	Prospective	Multicentric (United States)	To characterize early life growth trajectories and to determine if these trajectories are associated with Forced Expiratory Volume in 1 second at ages 6-7 years	Forced Expiratory Volume in 1 second (Percentage of predicted), Weight For Length, Body Mass Index	Subjects with Weight For Length and Body Mass Index always>50th percentile had the highest Forced Expiratory Volume in 1 second (Percentage of predicted) predicted at age 6-7 years, significantly higher than subjects whose Weight For Length and Body Mass Index was stable or decreased>10 percentile points

## Data synthesis

A total of 12,455 patients were assessed. Considering the start up period of each study, the age range of the patients was 0-15.3 years old. According to the study design, one was a cohort study,^12^ four were longitudinal studies^13^^-^ 15 ^,^ 17 and two were prospective studies.^16^^,^ 18

The selected studies used several growth and nutritional parameters. Among the nutritional and growth parameters, Body Mass Index (BMI) was evaluated in five studies,^12^^,^ 14 ^-^ 16 ^,^ 18 height alone was evaluated in two studies,^13^^,^ 15 height gain was evaluated in two studies,^13^^,^ 15 Weight For Age (WFA) was evaluated in two studies,^14^^,^ 18 Height For Age (HFA) was evaluated in two studies.^14^^,^ 16 The remaining parameters were evaluated each one in one study: weight alone,^14^ percent of height-appropriate Body Weight (%haBW),^12^ percentage of the Ideal Body Weight (%IBW),^12^ Height for Age *Z*-score (HAZ),^12^ Weight for Age *Z*-score (WAZ),^12^ Height-For-Age adjusted for Target Height (HFA/TH),^17^ Weight-For-Length and BMI percentiles (WFL-BMI)^18^ (Tables 1 and 2).

All studies used Forced Expiratory Volume in 1 second (FEV1 or FEV1% predicted) as a measure for pulmonary function and only one used forced vital capacity.^14^

Six studies assessed the relationship of growth and nutritional parameters with pulmonary function^12^^-^ 14 ^,^ 16 ^-^ 18 and only one study assessed the relationship of growth alone with pulmonary function.^15^

## Main results

Zemel et al.^12^ studied a large sample (968 children) from multiple care centers across the United States during a 4 year period. During the first 3 years of follow-up, the female patients declined in HAZ, whereas male patients increased HAZ. Both groups declined in this same parameter in the last year. There were differences in the pattern of longitudinal change in FEV1%, and the rate of decline was less in male patients. The authors found that HAZ was positively and significantly associated with FEV1%, as were WAZ and %haBW.

Konstan et al.^13^ examined, in 931 patients, the relative role of growth and nutritional status and the clinical evidence of lung disease from age 3-6 years in determining pulmonary function at 6 years of age. The results showed that mean WFA and HFA at both ages 3 and 6 years were below the 50th percentile for healthy children. The presence of signs and symptoms of lung disease at age 3 was only weakly associated with growth and nutritional categories in this same age. However, patients with lower growth and nutritional indexes at age 3 had lower pulmonary function at age 6, and this was evident for WFA and HFA, in which the differences in the percent predicted lung function values reached 15 and 12 points for the lowest and high WFA and HFA categories, respectively.

Peterson et al.^14^ prospectively examined data of 319 children from ages 6-8 years. Total weight change (kg/month) between the child's first and last visits was examined as a marker of cumulative growth during the 2 year period. In repeated measures regression analysis, FEV1 values did not vary significantly by height change within patients with high or low weight change. Neither the height nor change in the height from first observation were significantly associated with FEV1 values, but children who had a steady weight gain tended to experience greater increases in FEV1 than those who lose weight.

Assael et al.^15^ followed 163 patients in order to evaluate the relationship between linear growth and lung disease severity. This study showed a progressive loss of FEV1 in patients with mild and severe lung disease. In patients with severe disease, the pre-pubertal take-off and peak occurred later and at slightly lower velocity compared to patients with mild disease. Pubertal peak of severe patients occured about 8 months later than that of mild patients. Their peak velocity was lower by 1.3cm/year (vs. mild disease) and 2.0cm/year (vs. healthy subjects). When age, height and height velocity at all the four growth milestones were considered as a whole, the differences between patients with mild and severe disease were highly significant.

Yen et al.,^16^ in a prospective observational study, evaluated 3,142 CF patients and found that FEV1% was lower in the group of patients with WAP<10% at age 4 years than all other WAP groups, never reaching a FEV1>80% throughout the study period. This study also found that patients in the highest weight and height percentiles at age 4 had fewer pulmonary exacerbations, spent fewer days in the hospital, and had better survival at 18 years of age.

Woestenenk et al.,^17^ studying 156 children in a mean period of 7.4 years, did not find correlation between FEV1% and either weight or height, in a first cross sectional analysis. However, regardless of their initial WFA or WFH category, children who increased in weight had minor declines in FEV1% predicted from 6 to 10 years old. The decline in FEV1% slowed by 1.8% and 1.9%, respectively, for each unit of increase in WFA and WFH. The study did not find any association between FEV_1_ and either HFA or HFA/TH.

Sanders et al.^18^ prospectively studied 6,805 patients in the Cystic Fibrosis Foundation Patient Registry. Children with CF born between 1994 and 2005 followed from age ≤2 through 7 years were assessed according to changes in annualized WFL percentiles between ages 0 and 2 years and BMI percentiles between ages 2 and 6 years. The results of a multivariable linear regression model showed that subjects with WFL-BMI always>50th percentile had the highest FEV1% predicted at age 6-7 years, significantly higher than subjects whose WFL-BMI was stable or decreased>10 percentile points.

## Discussion

Studies for the management of CF patients have been contributing for the advance in the multidisciplinary care, aiming at the best treatment of this disease. Despite these improvements, pulmonary insufficiency remains as the main cause of death in CF and there are strong associations between growth and nutritional indices and lung function.^19^ However, most studies reporting these evidences are cross-sectional, with few evidences to support association between longitudinal changes in growth and nutrition with changes in lung function through life.

The study conducted by Assael et al.^15^ was the first one to demonstrate a clear association between early growth events and lung function in CF patients. This study showed a progressive loss of FEV1 in patients with mild and severe lung disease. In patients with severe lung disease, the pre-pubertal take-off and peak occurred later and at slightly lower velocity compared to patients with mild disease. Pubertal peak of severe patients occurred about 8 months later than that of mild patients. Their peak velocity was lower by 1.3cm/year (vs. mild disease) and 2.0cm/year (vs. healthy subjects). When age, height and height velocity at all the four growth milestones were considered as a whole, the differences between patients with mild and severe disease were highly significant. The main finding of this study is that low height velocity is an early manifestation of lung disease severity in CF.

Two studies^14^^,^ 17 did not find associations between changes in height and changes in FEV1, but these studies were conducted in shorter periods of time, and, differently from two studies previously mentioned,^15^^,^ 16 their analyses included children whose ages ranged from 2 to 10 years old. Thus, the influence of growth spurts could not be assessed. However, both studies point to an association between weight gain and better lung function.

Three studies^12^^,^ 13 ^,^ 18 reported that the improvement of both growth and clinical parameters in early life was associated with a better lung function 2-5 years later. Thus, it is suggested that growth and nutritional status in childhood may be strong predictors of the severity of lung disease later in life, which is reinforced by Yen et al.,^16^ who showed that patients in the highest weight and height percentiles at age 4 years had fewer pulmonary exacerbations, spent fewer days in the hospital, and had better survival at age 18 years.

Pulmonary function abnormalities, including decreased peak flow and lower vital capacity, forced vital capacity, and forced expiratory flow, occur in other conditions in which children are malnourished and have stunted growth.^20^^-^ 23 Nutritional interventions with malnourished patients showed that enhanced caloric intake resulted in increased weight and height gain velocities and improvements in other nutritional status measures, besides reduction in cases of pulmonary infections or slower deterioration in pulmonary function.^10^^,^ 24 ^-^ 26 Growth and nutrition are two intimately linked characteristics, so, since better nutrition is associated to better lung function in CF, maintaining a healthy nutritional status is important not only for nutrition and growth, but for lung function as well.^27^ Stephenson et al.^28^ studied a cohort of 909 CF patients. Subjects in the overweight and obese categories were older and presented better lung function. Within the underweight group, a 10% increase in BMI resulted in a 4% relative increase in FEV1 and individuals with a BMI in the adequate range had a 5% relative increase in FEV1. This was the first study to characterize changes in nutritional status overt time and to quantify the relation between nutrition and lung function across the spectrum of BMI categories. Regarding to improvement in growth, the evidences for clinical trials are also limited. Growth hormone therapy does not seem to improve lung function at a significant level.^29^

In summary, the studies highlight the importance of optimizing growth and nutritional status in CF patients by an aggressive nutritional intervention, and also by treatment of pulmonary disease, even in those with milder pulmonary disease. However, the relationship of longitudinal growth and lung function in CF is still unclear and must be better investigated. Controlled trials with therapies focused on the pulmonary disease are necessary to investigate its relationship with growth and nutritional status. Given the deterioration that lungs suffer with time in CF, long term studies are important to better characterize these parameters and to identify which factors are more deeply involved in this process, thus, appropriate interventions may be implemented. More sensitive measures for lung function, such as lung clearance index, are also necessary to better diagnose the severity of lung disease, mainly for the preschool age range.^30^ There are about 2000 mutations of the CFTR gene, distributed among six classes. Thus, studies of association among variables must, in the future, be structured for mutation classes that configure a more severe disease (classes I, II and III). The higher facility of access to medicines, attendance at reference centers, neonatal screening, early treatment for *Pseudomonas aeruginosa* infection, early intake of pancreatic enzymes, knowledge of polymorphisms and differential follow-up in adolescence and adulthood (mainly for the female gender) are factors that may equilibrate and modify the natural history of CF with regard to the pulmonary function decline, growth and nutrition of these patients.
